# The cyclooxygenase-expressing mesenchyme resists intestinal epithelial injury by paracrine signaling

**DOI:** 10.1186/s13619-023-00174-7

**Published:** 2023-08-14

**Authors:** Siting Wei, Meng Li, Wanlu Song, Jiaye Liu, Shicheng Yu, Yalong Wang, Mengxian Zhang, Huijun Du, Yuan Liu, Huidong Liu, Wei Fu, Baojie Li, Ye-Guang Chen

**Affiliations:** 1 The State Key Laboratory of Membrane Biology, Tsinghua-Peking Center for Life Sciences, School of Life Sciences, Tsinghua University, Beijing, 100084 China; 2 Guangzhou National Laboratory, Guangzhou, 510005 China; 3 Department of Thyroid and Parathyroid Surgery, West China Hospital, Sichuan University, Chengdu, Sichuan China; 4 Department of General Surgery, Peking University Third Hospital, Beijing, China; 5 Bio-X Institutes, Key Laboratory for the Genetics of Developmental and Neuro- Psychiatric Disorders, Ministry of Education, Shanghai Jiao Tong University, Shanghai, 200240 China; 6 School of Basic Medicine, Jiangxi Medical College, Nanchang University, Nanchang, 330031 China

**Keywords:** Colitis, Intestinal mucosal barrier, Mesenchymal stromal cells, Organoids, Prostaglandin E2

## Abstract

Paracrine signals play pivotal roles in organ homeostasis. Mesenchymal stromal cells (MSCs) play a key role in regulating epithelium homeostasis in the intestine while their paracrine effects are poorly characterized. Here, we identified prostaglandin E2 (PGE2) secreted by cyclooxygenase (COX)-expressing MSCs as a vital factor to maintain the intestinal mucosal barrier. We found that MSCs-induced organoid swelling through paracrine effect in vitro, a process due to enhanced water adsorption and is mediated by the COX-PGE2-EP4 axis. To further explore the regulatory effect of this axis on the intestinal epithelial barrier in vivo, we established the conditional knockout mouse model to specifically delete COX in MSCs and found that PGE2 reduction downregulated the gene *Muc2* and induced a gastric metaplasia-like phenotype. Moreover, PGE2 defects increased the susceptibility of intestinal epithelium to colitis. Our study uncovers the paracrine signaling of COX-expressing MSCs in intestinal mucosal barrier maintenance, providing a basis for understanding the role of mesenchymal cells in the pathophysiological function of the intestine.

**Supplementary Information:**

The online version contains supplementary material available at 10.1186/s13619-023-00174-7.

## Background

The intestine is a crucial compartment of the gastrointestinal tract that is responsible for nutrient uptake, protection against the microbiota and immune modulation (Beumer and Clevers 2021; Caruso et al. 2020; Hageman et al. 2020; McCarthy et al. 2020). The intestinal epithelium possesses a self-renewal ability to cope with constant challenges, which is driven by the Lgr5^+^ stem cells at the bottom of crypt that actively proliferate and differentiate into various functional cells to ensure the daily renewal of intestinal epithelium (Barker et al. 2007; Gehart and Clevers 2019). Alongside the epithelium, the underlying mesenchymal cells, immune cells and other cell types constitute a dynamic environment and provide signals to regulate the intestinal cell fate (Beumer and Clevers 2021).

Mesenchymal stromal cells (MSCs), which include myofibroblasts, pericytes, and several groups of stromal cells, are a source of soluble signals (Kinchen et al. 2018). For instance, they contribute to Bone Morphogenetic Protein (BMP) gradient establishment along the villus-crypt axis to maintain epithelial differentiation (Kosinski et al. 2007), and BMP gradient also contributes to zonated gene expression in enterocytes and goblets (Beumer et al. 2022). Certain subpopulations could activate Wnt signaling to support the stem cell niche (Stzepourginski et al. 2017). Intestinal MSCs could be genetically marked by platelet-derived growth factor receptor alpha (*Pdgfrα*), alpha-smooth muscle actin (*Acta2*), *Foxl1*, *Twist2* or *Gli1* (Aoki et al. 2016; Degirmenci et al. 2018; Gao et al. 2021; Jacob et al. 2022). In particular, Gli1^+^ MSCs located near the crypt base could secrete high Wnt2b to promote epithelial Wnt signaling and thus maintain intestinal homeostasis (Valenta et al. 2016). However, due to the heterogeneity of intestinal MSCs and the huge diversity of signaling cues derived from them, the paracrine effect of intestinal MSCs on the intestinal epithelium still remain poorly characterized.

Prostaglandin E2 (PGE2), as a microenvironment factor of intestinal epithelium, is critical for intestinal epithelium either in physiological or pathological conditions. PGE2 regulates the fluid and electrolyte homeostasis of intestinal epithelium by increasing cyclic AMP (cAMP) and thus activating cystic fibrosis transmembrane conductance regulator (CFTR), which functions as a Cl^−^ channel as well as a HCO3^−^ channel (Fujii et al. 2016). After injury, PGE2 promotes wound-associated cell differentiation and wound repair in the intestine (Miyoshi et al. 2017). Fibroblast-derived PGE2 drives the expansion of Sca-1^+^ reserve-like stem cells by the Hippo pathway effector YAP to initiate intestinal tumor (Roulis et al. 2020).

Inflammation bowl disease (IBD), including two major forms termed ulcerative colitis (UC) and Crohn’s disease (CD), is a chronic inflammation disorder caused by the interplay of environmental exposures, immune responses and various other factors (Adolph et al. 2022; Ananthakrishnan et al. 2022). These factors cause the continuous damage to the intestinal epithelium, and yet there is no curative treatment for this chronic disease (Villablanca et al. 2022). In the active inflammatory tissues of IBD patients, PGE2 production is increased, which may be induced by upregulation of COX2, one of two key enzymes in the synthesis of prostaglandins using arachidonic acid as the substrate (Rampton and Hawkey 1984; Wang and Dubois 2010). The COX2 is expressed in multiple cell types of the intestine, including activated macrophages, mast cells, epithelial cells and others (Smith et al. 2000). Nonsteroidal anti-inflammatory drugs (NSAIDs) exert their anti-inflammatory ability by inhibiting COX2 (Zaman et al. 2022). Although the evidence remains contradictory, NSAIDs are not recommended for IBD treatment due to its adverse effect, which may promote IBD pathogenesis or progression (Sandborn and Hanauer 2003; Takeuchi et al. 2006). As one of the major sources of PGE2, MSCs may be involved in this process. However, COX expression and function in intestinal MSCs especially under inflammatory conditions are not well studied.

In this study, we investigated the paracrine effect of intestinal MSCs on intestinal epithelial cells. We discovered that COX-expressing MSCs secreted PGE2, which induced organoid swelling due to increased water adsorption via its receptor EP4. Deletion of mesenchymal COX suppressed this process and impaired the expression of *Muc2* and aggravated DSS-induced colitis in vivo. These results highlight a critical role of COX-expressing MSCs in maintaining intestinal homeostasis and reducing injury via provision of PGE2 in a paracrine manner.

## Results

### MSCs induce swelling of intestinal organoids via paracrine effect

**Fig. 1:**
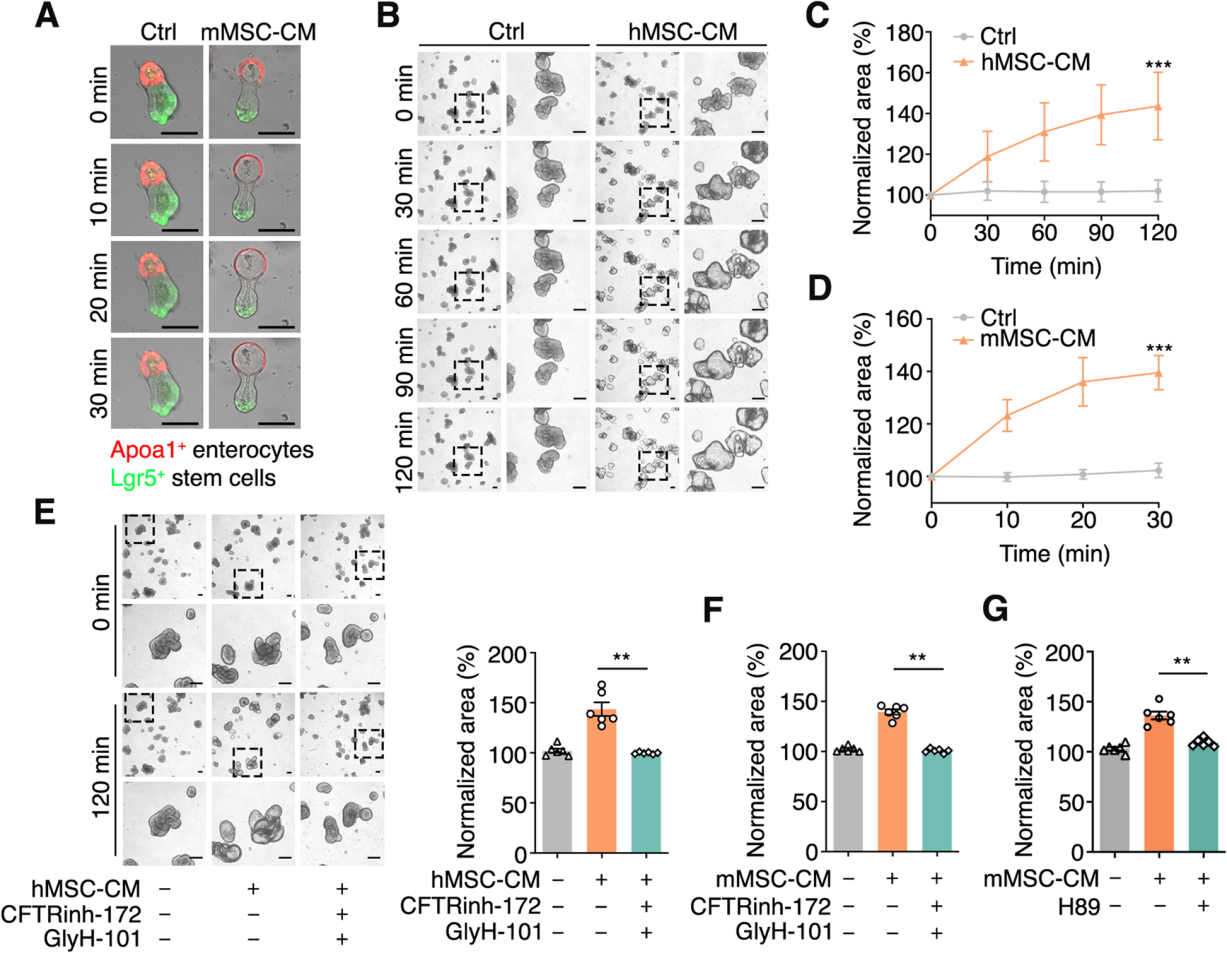
MSC-CM induces the intestinal organoid swelling by activating CFTR. **A** Time-lapse imaging of mouse *Apoa1-mCherry; Lgr5-EGFP* organoids with mouse small intestinal MSC-CM (mMSC-CM) stimulation. Scale bar, 100 μm. **B** Time-lapse imaging of human ileum organoids with human ileum MSC-CM (hMSC-CM) stimulation. Scale bar, 100 μm. **C**, **D** Quantification of surface area of human (**C**) or mouse (**D**) small intestinal organoids in response to the MSC-CM stimulation. The normalized area is the proportion of organoid surface area relative to the surface area at t = 0. **E** Time-lapse imaging and surface area quantification of CFTRinh-172 and GlyH-101-treated human ileum organoids with hMSC-CM stimulation. The human small intestinal organoids were preincubated with 50 μM CFTRinh-172 and 50 μM GlyH-101 (CFTR inhibitors) for 3 h before stimulation. Scale bar, 100 μm. **F** Quantification of surface area of CFTRinh-172 and GlyH-101-treated mouse small intestinal organoids with mMSC-CM stimulation. The mouse small intestinal organoids were preincubated with 50 μM CFTRinh-172 and 50 μM GlyH-101 (CFTR inhibitors) for 3 h before stimulation. **G** Surface area quantification of H89-treated mouse small intestinal organoids after mMSC-CM stimulation. The mouse small intestinal organoids were preincubated with 10 μM H89 (PKA inhibitor) for 6 h before stimulation. Data represent mean ± SD of three independent experiments. **P < 0.01, ***P < 0.001, unpaired two-tailed *t*-test (**C**, **D**), one-way ANOVA (**E**, **F**, **G**)

To investigate the intestinal mesenchymal paracrine effect on the epithelium, we generated organoids from the crypts of the small intestine isolated from *Apoa1-mCherry* mice and then treated the organoids with the conditional medium from MSCs (MSC-CM) from mouse small intestine (Fig. S1A). We found that MSC-CM induced the rapid swelling of the organoids with Apoa1^+^ enterocytes (Figs. 1A, S1B and Movie S1). The swelling was also observed in human organoids treated with human MSC-CM (Fig. 1B). To quantify the swelling of organoids, we calculated the change of two-dimensional area of organoids after MSC-CM addition and found that the swelling was increased in a time-dependent manner. Both human and mouse organoids showed a rapid swelling response lasting for at least 120 min and 30 min, respectively (Fig. 1C, D). Interestingly, the conditional medium of NIH-3T3 cells (NIH3T3-CM) could also induce organoid swelling, but the one of L cells (L-CM) did not (Fig. S1C, D).

Forskolin, which promotes the intracellular cAMP production and thereby activates the Cl^−^ channel CFTR, was shown to induce rapid swelling of organoids (Dekkers et al. 2013). To examine whether the Cl^−^ channel CFTR was involved in this process, we treated organoids with CFTR inhibitors, CFTRinh-172 and GlyH-101, and found that the MSC-CM-induced organoid swelling was blocked (Figs. 1E, F and S1E). Protein kinase A (PKA), which is activated by cAMP, is required for the activation of CFTR channel (Liu et al. 2017). Consistently, the PKA inhibitor H89 inhibited the organoid swelling (Figs. 1G, S1F). Taken together, these results indicate that MSCs may secrete factors to induce organoid swelling in a CFTR-dependent manner.

### The COX-PGE2-EP4 axis contributes to the MSC-CM-induced swelling of intestinal organoids

**Fig. 2:**
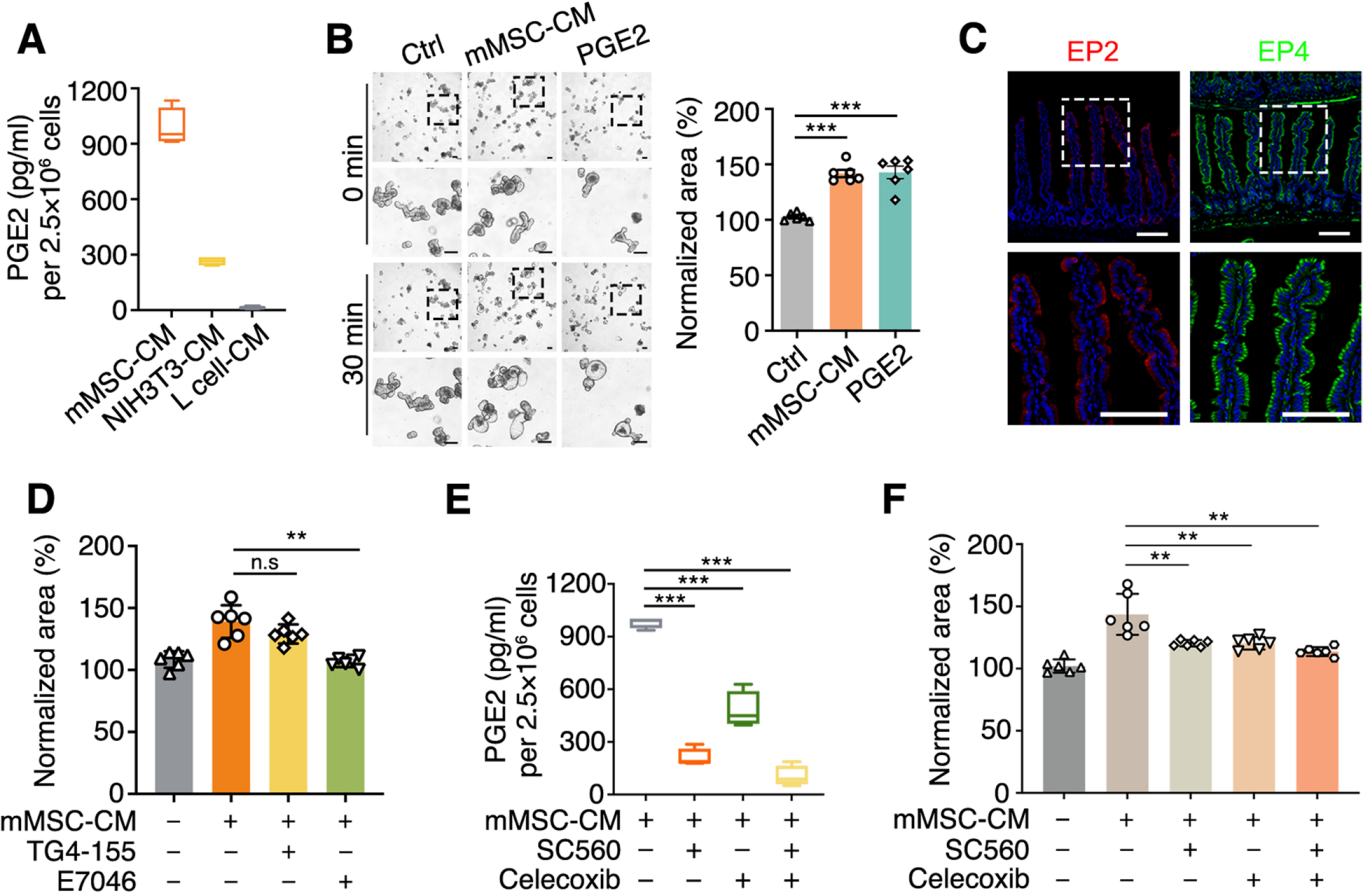
The COX-PGE2-EP4 axis is involved in the MSC-CM-induced organoid swelling. **A** The PGE2 concentration of mMSC-CM, NIH3T3-CM or L cell-CM, quantified by ELISA assay. **B** Time-lapse imaging and surface area quantification of small intestinal organoids with mMSC-CM or PGE2 (7 nM) stimulation. Scale bar, 100 μm. **C** Immunofluorescent staining of EP2 and EP4 in mouse small intestine. Scale bar, 100 μm.** D** Quantification of surface area of E7046 or TG4-155-treated mouse small intestinal organoids with mMSC-CM stimulation. The mouse small intestinal organoids were preincubated with 10 μM E7046 (EP4 inhibitor) or 10 μM TG4-155 (EP2 inhibitor) for 72 h before stimulation. **E** The PGE2 concentration of SC560 or Celecoxib-treated mMSC-CM, quantified by ELISA assay. **F** Surface area quantification of mouse small intestinal organoids with stimulation using SC560 or Celecoxib-treated mMSC-CM. The mMSCs were preincubated with 20 nM SC560 (COX1 inhibitor), 150 nM Celecoxib (COX2 inhibitor), or both for 48 h, then the CM was collected for stimulation. Data represent mean ± SD of three independent experiments. **P < 0.01, ***P < 0.001, one-way ANOVA (**B**, **D**, **E**, **F**)

To identify the factors in MSC-CM that induced the swelling, mouse MSC-CM was subjected to Liquid Chromatography with Tandem Mass Spectrometry (LC–MS). NIH3T3-CM and L-CM was used as the positive and negative control, respectively. We found PGE2 in mouse MSC-CM and NIH3T3-CM, but not in the L-CM (Fig. S2A). The level of PGE2 in these three kinds of CM was confirmed by ELISA (Fig. 2A). More importantly, exogenous PGE2 also induced organoid swelling at the same concentration in mouse MSC-CM (Fig. 2B).

PGE2 signals by binding to one of its four receptor subtypes, including EP1 (encoded by *Ptger1*), EP2 (encoded by *Ptger2*), EP3 (encoded by *Ptger3*), and EP4 (encoded by *Ptger4*). To determine which receptor mediates PGE2 action, we analyzed the expressions of *Ptger1*, *Ptger2*, *Ptger3* and *Ptger4* in mouse intestinal epithelium through single-cell RNA sequencing (scRNA-seq) and found that *Ptger4* was at the highest expression in the epithelial cells (Fig. S2B). The EP4 expression was confirmed by immunostaining (Fig. 2C). When the mouse intestinal organoids were treated with specific inhibitors for EP2 (TG4-155) and EP4 (E7046), only the EP4 inhibitor blocked the mouse MSC-CM-induced swelling (Figs. 2D, S2C), indicating that EP4 is the major functional receptor to mediate PGE2-induced swelling in mouse small intestine.

COX1 and COX2 are involved in the PGE2 synthesis (Clasadonte et al. 2011). When mouse MSCs were pretreated with the COX1 selective inhibitor SC560 and the COX2 selective inhibitor celecoxib, the PGE2 level in MSC-CM was decreased, showing impaired ability to induce organoid swelling (Figs. 2E, F and S3A). This phenomenon was also observed in human organoids (Fig. S3B-D). The nonsteroidal anti-inflammatory drugs aspirin and indomethacin can inhibit COX activity (Flower 2003; Warner et al. 1999). The pretreatment of hMSCs with aspirin or indomethacin could also block the MSC-CM-induced swelling of human intestine organoids (Fig. S3E, F). Furthermore, the PGE analogue misoprostol could partially induce organoid swelling even in the presence of the COX inhibitors-treated human MSC-CM (Fig. S3E, F). The data together suggest that MSC-CM-induced swelling is mediated by the COX-PGE2-EP4 axis.

### COX deletion in Gli1^+^ MSCs abolishes the MSC-CM-induced organoid swelling

**Fig. 3:**
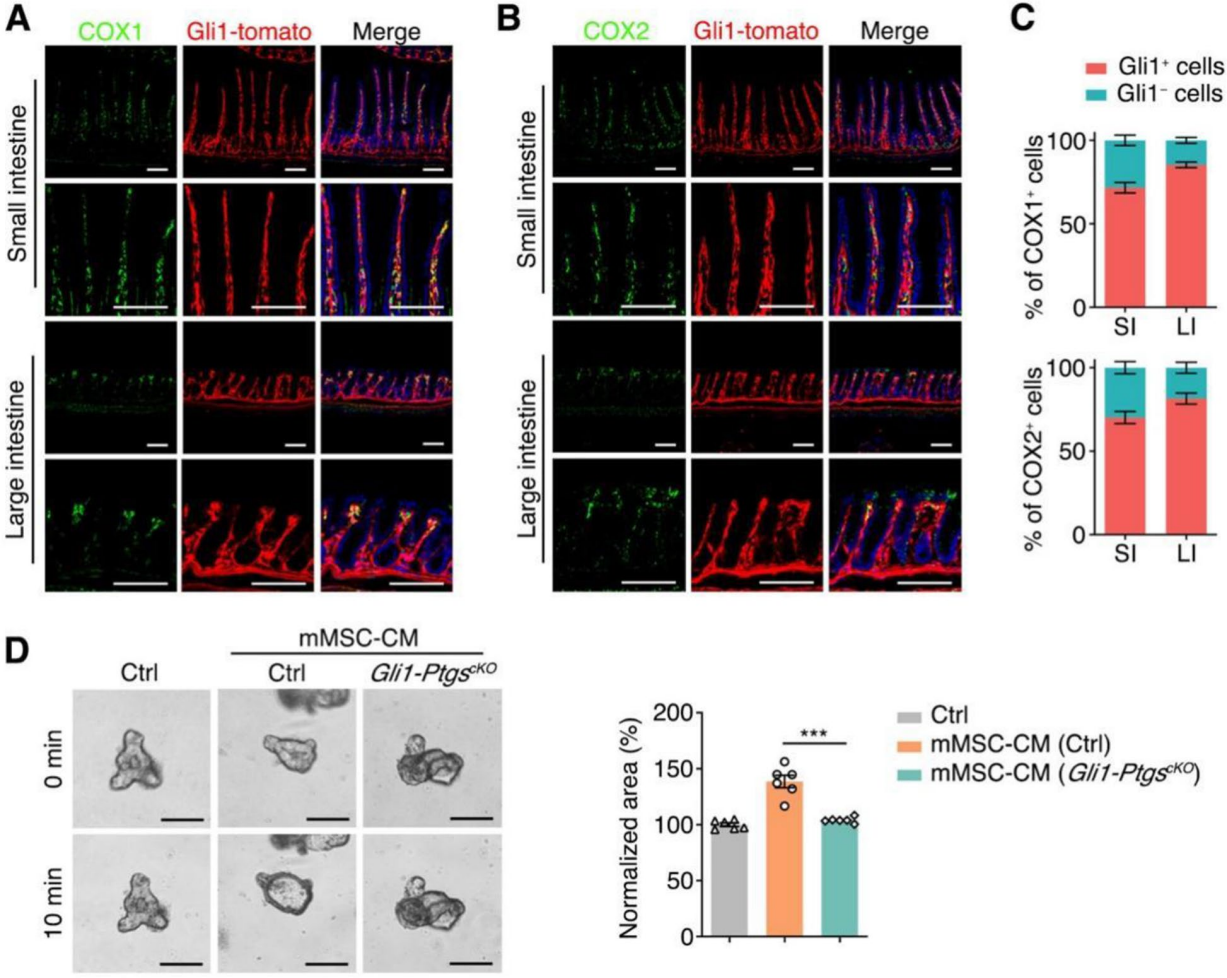
COX deletion inhibits the MSC-CM-induced organoid swelling. **A**, **B** Representative images of COX1 (**A**) or COX2 (**B**) distributions in Gli1-tomato cells in mouse small and large intestine. **C** Proportions of Gli1^+^ cells in COX1^+^ or COX2^+^ cells in mouse small (SI) and large intestine (LI). **D** Time-lapse imaging and surface area quantification of small intestinal organoids with stimulation using mMSC-CM from Ctrl or *Gli1-Ptgs*^*cKO*^ mice. Scale bar, 100 μm. Data represent mean ± SD of three independent experiments. ***P < 0.001, one-way ANOVA (**D**)

To validate the role of the COX-PGE2-EP4 axis in vivo, we first examined COX expression. The scRNA-seq revealed the expression of the COX1-encoding gene *Ptgs1* and the COX2-encoding gene *Ptgs2* in the intestinal MSCs (Fig. S4A), which was confirmed by the immunofluorescence staining (Fig. 3A, B). Intriguingly, *Ptgs1* expression was not overlapped with *Ptgs2* (Fig. S4B), so we simply defined MSCs expressing either *Ptgs1* or *Ptgs2* (or both) as the COX-expressing MSCs (the Plus cluster), and the rest as the Minus cluster (Fig. S4C). We found that the COX-expressing MSCs accounted for half of total intestinal MSCs (130/257) (Fig. S4D).

*Gli1* has been reported to be a marker of MSCs in mouse intestine (Degirmenci et al. 2018). The immunofluorescence analysis of *Gli1-Cre*^*ERT2*^*; Rosa26-tdTomato* mice revealed that most COX^+^ cells were Gli1-tdTomato^+^ cells (Fig. 3A-C). Therefore, to investigate the role of COX in MSCs, we crossed *Ptgs1*^*flox/flox*^ and *Ptgs2*^*flox/flox*^ mice with *Gli1-Cre*^*ERT2*^*; Rosa26-tdTomato* mice and generated *Gli1-Ptgs*^*cKO*^ mice, in which both COX1 and COX2 were knocked out in Gli1^+^ MSCs (Fig. S5A). Five-day tamoxifen administration led to nearly complete deletion of COX1 and COX2 in MSCs within one month (Fig. S5B). Then, the Gli1^+^ MSCs were isolated from *Gli1-Ptgs*^*cKO*^ mice by fluorescence-activated cell sorting (FACS) and were cultured in vitro. The CM from these cells lost the ability to stimulate the organoid swelling (Fig. 3D). These results indicate that COX1 and COX2 in the Gli1^+^ MSCs are critical to the MSC-CM-induced organoid swelling.

### COX-expressing MSCs support goblet cell differentiation

**Fig. 4:**
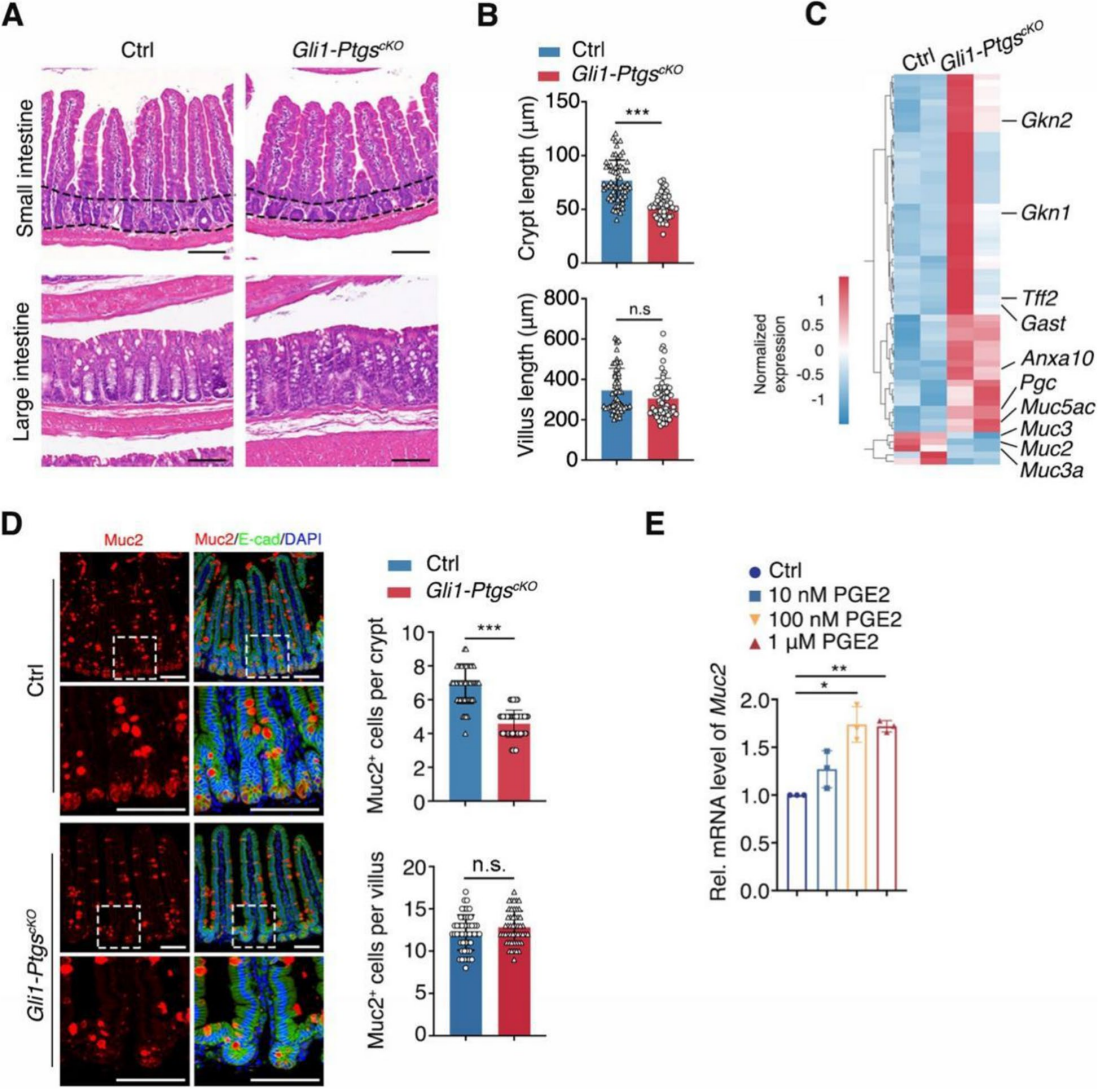
Loss of PGE2 dampens the *Muc2* expression in mouse intestinal epithelium. **A** Representative structures of *Gli1-Ptgs*^*cKO*^ (*Gli1-CreER; Ptgs1*^*flox/flox*^*; Ptgs2*^*flox/flox*^) and Ctrl (*Ptgs1*^*flox/flox*^*; Ptgs2*^*flox/flox*^) mouse small and large intestine indicated by H&E staining. The dashed lines indicate the crypt region of small intestine. Scale bar, 100 μm. **B** The crypt and villus length of *Gli1-Ptgs*^*cKO*^ and Ctrl mouse small intestine. **C** Heatmap showing differential expressed genes of small intestinal crypts between *Gli1-Ptgs*^*cKO*^ and Ctrl mice, analyzed from bulk RNA-seq. **D** Immunofluorescent staining of *Muc2* and quantification of Muc2^+^ cell number of *Gli1-Ptgs*^*cKO*^ and Ctrl mouse small intestine. Scale bar, 100 μm. **E** *Muc2* expressions of organoids treated with PGE2, as revealed by RT-PCR analysis. Data represent mean ± SD of three independent experiments. *P < 0.05, **P < 0.01, ***P < 0.001, n.s. not significant, unpaired two-tailed *t*-test (**B**, **D**), one-way ANOVA (**E**)

Since COX expression in MSCs was critical for MSCs to induce the epithelial swelling in vitro, we next addressed the function of COX-expressing MSCs in vivo. Since CFTR activation induced by PGE2 is crucial for the fluid secretion of the intestine, we first measured the stool water content of *Gli1-Ptgs*^*cKO*^ mice. However, one month after Cre induction, COX deletion appeared to have minimal effects on the stool water content (Fig. S5C), perhaps due to the existence of other MSCs not labeled by Gli1 that could still secrete PGE2. Moreover, *Gli1-Ptgs*^*cKO*^ mice showed normal intestinal length and body weight (Fig. S6A, B). Intriguingly, shorter crypt length of the small intestine was observed in *Gli1-Ptgs*^*cKO*^ mice (Fig. 4A, B). To explore the gene profiling changes of shorter crypts, the control (Ctrl) and mutant crypts were targeted for bulk RNA-seq. The results revealed that *Muc2* expression was decreased in the mutant group (Fig. 4C), which was validated by immunofluorescent staining (Fig. 4D), indicating that COX deletion leads to reduction of goblet cells. We also examined other intestinal cell lineages and found that Lyz1^+^ Paneth cells were decreased (Fig. S6C), while no changes were detected in Chga^+^ enteroendocrine cells (Fig. S6D) and Olfm4^+^ stem cells (Fig. S6E). To further investigate whether PGE2 regulates *Muc2* expression, small intestinal organoids were cultured in the absence or presence of PGE2. Consistent with the in vivo results, PGE2 could enhance *Muc2* expression (Fig. 4E). Mucin plays an important role in protecting the gastrointestinal epithelium from injury (Png et al. 2010). Through analyzing the Gene Expression Omnibus (GEO) DataSets, we observed increased *Muc2* expression in gut biopsies from IBD patients compared to healthy individuals (Fig. S6F). This is consistent with the RNA-seq analysis of deferentially expression genes between control group and *Gli1-Ptgs*^*cKO*^ group, showing that *Muc2* was a top downregulated gene in the volcano plot (Fig. S6G). The upregulation of MUC2 in IBD is likely a compensatory mechanism for damages in the epithelium barrier.

We also detected the upregulated expression of *Anxa10*, *Tff2*, *Gkn1*, *Gkn2*, *Gast*, *Muc5ac* and *Pgc* (Figs. 4C, S6G), which are all highly expressed in the gastric mucosa (Busslinger et al. 2021; Huebner et al. 2023; Kim et al. 2022; Seidlitz et al. 2019). This phenotype is similar to the gastric metaplasia characterized by the appearance of epithelial cells with the gastric identity in the duodenum (Shaoul et al. 2000). The prevalence of gastric metaplasia has been reported in intestinal inflammatory diseases including CD and non-specific chronic duodenitis (Fitzgibbons et al. 1988; Wright et al. 1990), and the expression of MUC5AC was detected in UC (Bara et al. 2003). We speculated that the gastric metaplasia-like phenotype resulting from COX1 and COX2 depletion in MSCs might reflect an abnormality of the intestinal epithelium prone to inflammation. In addition, the cell viability of MSCs derived from the *Gli1-Ptgs*^*cKO*^ group was decreased compared to that of control cells (Fig. S7).

### COX deficiency in MSCs makes the epithelium more vulnerable to DSS treatment

**Fig. 5:**
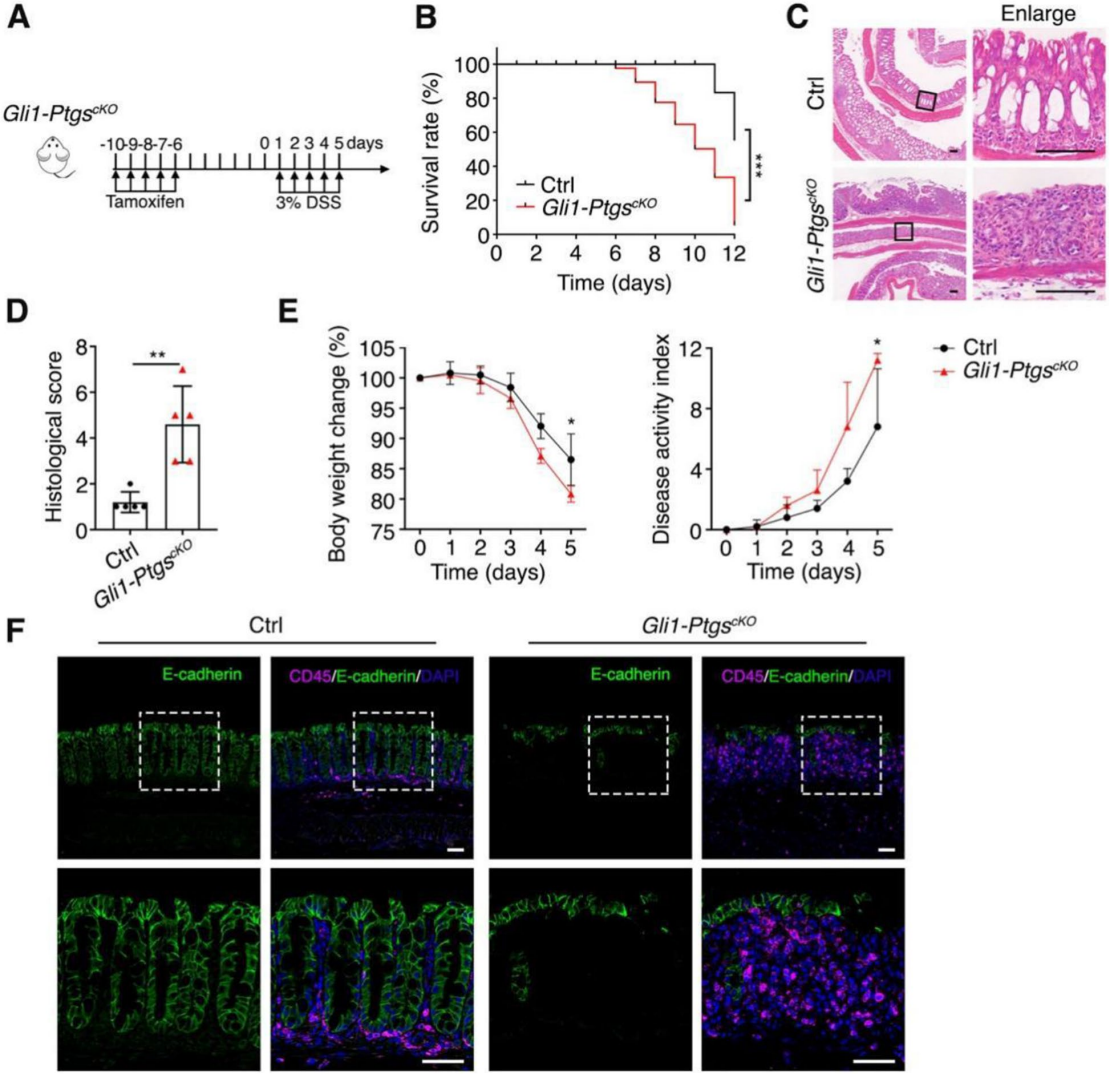
COX deletion aggravates DSS-induced epithelial injuries in colon. **A** Schematic diagram showing the timepoint for tamoxifen injection and DSS treatment. **B** The survival rate of *Gli1-Ptgs*^*cKO*^ and Ctrl mice (*n* = 6 per genotype). **C** Representative structures of *Gli1-Ptgs*^*cKO*^ and Ctrl mouse colon after DSS treatment indicated by H&E staining. Mice treated with 3% DSS for 5 days followed by 2-day water feeding were sacrificed. Scale bar, 100 μm. **D** Histological score of *Gli1-Ptgs*^*cKO*^ and Ctrl mice after DSS treatment (*n* = 5 per genotype). **E** Body weight and disease activity index of *Gli1-Ptgs*^*cKO*^ and Ctrl mice after DSS treatment (*n* = 5 per genotype). **F** Immunofluorescent staining of CD45 in *Gli1-Ptgs*^*cKO*^ and Ctrl mouse colon after DSS treatment. Scale bar, 100 μm. Data represent mean ± SD of three independent experiments. *P < 0.05, **P < 0.01, Log-rank test (**B**), unpaired two-tailed *t*-test (**D**), Friedman test (**E**)

The decreased *Muc2* expression and the gastric metaplasia-like phenotype following COX deletion suggest that the epithelium might be more susceptible to intestinal injury and inflammation. Since PGE2 is a key mediator of inflammation in IBD, we next investigated the role of COX-expressing MSCs in the dextran sulphate sodium (DSS)-induced colitis mouse model. After treated with 3% DSS for 5 days (Fig. 5A), and *Gli1-Ptgs*^*cKO*^ mice exhibited a significant lower survival rate (Fig. 5B). The colon of *Gli1-Ptgs*^*cKO*^ mice lost the crypt-villus structure with higher histological score (Fig. 5C, D). The disease activity index (DAI) is commonly used to evaluate the grade and extent of DSS-induced colitis (Kihara et al. 2003). As shown in Fig. 5E, *Gli1-Ptgs*^*cKO*^ mice exhibited quicker body weight loss and higher DAI score with earlier occurrence of digestive tract symptoms such as diarrhea and bleeding. Moreover, more severe colonic epithelium injury was observed in *Gli1-Ptgs*^*cKO*^ mice at day 5 after DSS treatment, accompanied by the increase of CD45^+^ leukocyte infiltration (Fig. 5F) and *Muc2* deduction (Fig. S8), indicating a strong inflammatory response. These data together suggest that expression of COX1 and COX2 in MSCs is critical to the epithelium maintenance upon injury.

## Discussion

Emerging evidence indicates that intestinal MSCs provide not only structural support but also signaling molecules such as Wnt, BMP, BMP antagonists and cytokines that regulate epithelial cell fate determination (Gehart and Clevers 2019). Certain types of intercrypt mesenchymal cells have been reported to supply niche signals to stem cells: Pdgfrα^+^ MSCs as the source of Wnts and R-spondin 3 (Greicius et al. 2018), Gli1^+^ MSCs as the source of Wnt2b (Degirmenci et al. 2018), and CD34^+^GP38^+^Acta2^−^ MSCs as the source of Wnt2b, R-spondin 1 and Gremlin 1 (Stzepourginski et al. 2017). However, the paracrine effects of intestinal MSCs on the intestinal epithelium still remain largely unexplored considering the large diversity and heterogeneity of MSCs. In this study, we identified a subpopulation of COX-expressing MSCs that promote intestinal organoid swelling via PGE2 secretion to activate CFTR, support the *Muc2* expression of intestinal epithelium, and confer the epithelium resistance to injury in the DSS-induced colitis model.

CFTR functions as an anion channel and is critical for fluid and electrolyte homeostasis, and its mutations result in cystic fibrosis that affects the respiratory, digestive, endocrine, and reproductive system (Polgreen and Comellas 2022). Forskolin treatment on intestinal organoids could cause the opening of CFTR channel, thus leading into the organoid swelling due to water absorption (Dekkers et al. 2013). Our study revealed that PGE2 produced by COX-expressing MSCs could regulate CFTR channel activity in organoids. However, there is no abnormal fluid secretion of intestinal epithelium in *Gli1-Ptgs*^*cKO*^ mice. The reason may be that PGE2 is also produced by other Gli1^−^ cells, or from other sources, such as macrophages that could produce PGE2 to promote Wnt signaling of intestinal stem cells (Zhu et al. 2022).

We found that *Muc2* expression was significantly downregulated in *Gli1-Ptgs*^*cKO*^ mice. *Muc2* can protect the intestine epithelium from injury (Png et al. 2010), indicating that PEG2-generating MSCs may contribute to the integrity of the intestinal barrier via regulating Muc2 production. Indeed, depletion of COX1 and COX2 in Gli1^+^ MSCs resulted in more severe intestinal injury after DSS administration. It is consistent with the report that dual inhibition of COX1 and COX2 could lead to small intestinal ulcers, as mice treated with SC560 and celecoxib for 24 h displayed small bowel damage (Greicius et al. 2018). However, how depletion of COX1 and COX2 in Gli1^+^ MSCs leads to downregulation of Muc2 and whether the PKA-cAMP-CFTR axis is involved in this process are unclear.

We also observed an elevated expression in the intestinal epithelium of genes characteristic to the gastric mucosa upon COX ablation in MSCs. Such aberrant shift in expression pattern resembles gastric metaplasia found in patients with intestinal inflammatory diseases associated with antral *C. pylori* infection or serve as a defense mechanism for mucosal ulceration (Fitzgibbons et al. 1988; Wright et al. 1990). It is possible that the gastric metaplasia-like program induced by mesenchymal COX deletion might prime the intestinal epithelium for inflammation, and further investigation is required for clarifying its biological implications.

It has been reported that inhibition of COX2/PGE2 confers anti-inflammation in many conditions (Bertolini et al. 2001; Cui and Jia 2021). However, we found that COX deficiency in MSCs makes the epithelium more vulnerable to DSS-induced colitis. Our data are in agreement with other reports. For instance, human umbilical cord blood-derived MSCs could reduce colitis by producing PGE2 (Kim et al. 2013), and ablation of the TLR4-p38MAPK-Cox2 pathway in MSCs aggravated colitis development (Gao et al. 2021). These results are consistent with the observation that NSAIDs were commonly used for inflammation treatment, but they sometimes cause IBD relapse and gastrointestinal complications (Wang et al. 2021). The role of PGE2 in colitis may be context-dependent, perhaps due to the complex immune responses. The underlying mechanism await further investigation.

## Methods

### Animals

*Apoa1-mCherry* mice were commercially generated by our group through Gem Pharmatech (Nanjing); *Rosa26-Loxp-STOP-Loxp-tdTomato* mice were obtained from Jackson Laboratory; *Ptgs1*^*flox/flox*^ mouse strain was obtained from Gem Pharmatech. Both male and female mice were back-crossed into the C57BL/6 genetic background. For Cre-mediated recombination, mice were intraperitoneally injected (100 mg/kg) with tamoxifen (Sigma) for 5 consecutive days. All mice were housed in the pathogen-free Laboratory Animal Facility at Tsinghua University. All animal studies were under the approval of the Institutional Animal Care and Use Committee of Tsinghua University.

### Stool collection

Mice were placed in a separate clean cage and fecal pellets were collected immediately in sealed tubes. To measure stool water content the fecal pellets were dried at 65℃ for 48 h and the stool water content was calculated as follows: (wet weight—dry weight)/wet weight.

### Mouse intestinal crypt isolation and organoid culture

Mouse intestinal crypts were isolated and cultured as previously described (Qi et al. 2017; Sato et al. 2011). Isolated crypts were embedded in Matrigel (BD) and seeded on 48- or 24-well plate. After polymerization, isolated crypts were cultured in culture medium (Advanced DMEME/F12, Thermo Fisher) supplemented with Penicillin/Streptomycin (Thermo Fisher), GlutaMAX-I (Thermo Fisher), N2 (Thermo Fisher), B27 (Thermo Fisher) and N-acetylcysteine (Sigma-Aldrich), EGF (50 ng/ml, Peprotech), Noggin (100 ng/ml, Novoprotein) and R-spondin1 (500 ng/ml, Novoprotein). The culture medium was added and refreshed every 3 days.

### Human intestinal crypt isolation and organoid culture

Human intestinal crypts were isolated and cultured as previously described (Wang et al. 2020). Isolated crypts were cultured in culture medium (Advanced DMEME/F12) supplemented with Penicillin/Streptomycin, GlutaMAX-I, N2, B27, N-acetylcysteine, EGF (50 ng/ml,), Noggin (100 ng/ml) and R-spondin1 (500 ng/ml), CHIR-99021 (5 μM, Selleck), A83-01 (0.5 μM, MCE), SB202190 (10 μM, Selleck), Gastrin (1 nM, Tocris), Y27632 (10 μM, Selleck), PGE2 (2.5 μM, Selleck), and Nicotinamide (10 mM, Sigma-Aldrich). The culture medium was added and refreshed every 3 days.

### Intestinal MSC isolation and culture

The fat and feces of intestinal tissue were removed, and tissue was cut into small pieces (5 mm) and incubated in 1 mM DTT (sigma) and 30 mM EDTA (Beyotime) for 10 min at 250 rpm and then vortexed vigorously for 2 min. Then, the tissues were cut up and dissociated in RPMI-1640 containing 150 μg/mL DNase I (Promega), Collagenase VIII (Sigma, 100 U/mL for mouse, 2000 U/mL for human) at 37 ℃ for 20 min. MSCs were plated on 6 cm dish to grow and cultured in MSC culture medium (αMEM, Gibco) supplemented with 10% FBS (ExCell Bio) and Penicillin/Streptomycin.

### PGE2 concentration determination

MSCs were seeded in 10 cm dish. At 80–90% confluence, cells were washed with PBS for twice and the medium was replaced with 3 mL serum-free DMEM medium. After 24 h, the cell number was counted, and the medium was collected and centrifuged for 5 min at 1200 g to remove cell debris. The PGE2 concentration of MSC-CM was measured by using a Prostaglandin E2 Express ELISA Kit (Cayman Chemical, 500,141) according to manual instructions.

### Immunofluorescent staining

The intestine was isolated and fixed with 4% formaldehyde solution, then embedded in OCT. The cryosections were prepared. Then, the sections were permeabilized for 15 min with 0.1% Triton X-100 on ice and blocked in 5% BSA for 1 h at room temperature. The sections were then incubated overnight with the primary antibody at 4 ℃. Finally, the fluorescein-labelled secondary antibodies were applied for 1 h at room temperature. The following antibodies were used: rabbit anti-COX1 (Abcam, 1:200); rabbit anti-COX2 (Abcam, 1:200); mouse anti-PTGES (Santa Cruz, 1:50); rabbit anti-Muc2 (Abcam, 1:300); rabbit anti-Lyz (Abcam, 1:300); rabbit anti-Chga (Abcam, 1:300); rabbit anti-Olfm4 (CST, 1:300).

### Immunoblotting

Protein lysates were prepared from intestinal MSCs. These assays were performed as previously described (Qi et al. 2017). The following primary antibodies were used: rabbit anti-COX1 (Abcam, 1:1000); rabbit anti-COX2 (Abcam, 1:1000); GAPDH (Santa Cruz, 1:1000).

### Single-cell RNA seq

Single-cell geneexpression data of mice duodenum, jejunum, ileum, colon and rectum were generated using Chromium Single Cell 3’ Reagent Kits by 10X Genomics (https://support.10xgenomics.com/single-cell-gene-expression/software/downloads/latest). The reads were aligned to the mouse reference genome (mm10) using the Cellranger toolkit (v4.0.0). After filtering of low-quality cells with, UMI count matrices of single cells were retained for the subsequent analyses using R package Seurat. Unsupervised cell-clustering analysis was carried out and 18 unique cell clusters were identified, after differentially expressed genes (DEGs) analysis, specifically marking genes were calculated and cell types of each cluster were defined by canonical marker genes. Mesenchymal cells of large intestine were extracted and the co-expression of *Ptgs1* and *Ptgs2* were illustrated using UMAP (Uniform Manifold Approximation and Projection).

### Bulk RNA-seq

The crypts, villus and MSCs were isolated from mouse intestine respectively. Then RNA of these cells was extracted by RNeasy mini kit (74,104, Qiagen) as manuals. cDNA libraries were conducted with the Ovation RNA-Seq System V2 kit (NuGEN) and subjected to high-throughput sequencing on an Illumina Novaseq PE150 platform. RNA-seq was carried out with two biological replicates. Sequencing reads were aligned to mouse genome reference (mm10) using STAR (Spliced Transcripts Alignment to a Reference) with default parameter. Differential expression analysis was performed using the R package DESeq2 based on gene counts data. Differentially expressed genes were identified with Log_2_FoldChange absolute value > 1 and *p* value < 0.01.

### DSS-induced colitis

Acute colitis model was established by administering 8-week-old male C57BL/6 mice with 3% DSS (MP biomedicals) for 5 days. Body weight was monitored every day. The colitis disease activity index (DAI) was calculated daily for each mouse on the basis of weight loss, stool consistency and rectal bleeding, following a previously reported protocol (Gao et al. 2021). Histological score was calculated at day 5, as previously reported (Obermeier et al. 1999).

### RT-PCR analysis

mRNA was extracted from cells or tissue samples with Trizol (Thermo Fisher) as previously described (Qi et al. 2017). For RT-PCR analysis, the following primers were used: Gapdh, 5’-AAGAAGGTGGTGAAGCAG-3’ and 5’- TCATACCAGGAAATGAGC-3’; Muc2, 5’-TGTGGTCTGTGTGGGAACTTTG-3’ and 5’- GCTTACATCTGGGCAAGTGGAA-3’.

### Statistical analysis

Statistical analysis was performed with Graphpad Prism software. All data were represented as mean ± SD. Unpaired two-tailed t-test, one-way ANOVA, and Friedman test analysis was used to compare differences as indicated in the figure legends. n. s: not significant, ∗: *p* value < 0.05, ∗∗: *p* value < 0.01, ∗∗∗: *p* value < 0.001.

### Supplementary Information

**Additional file 1: Supplemental Movie 1.** Video shows mMSC-CM-induced swelling of mouse small intestinal organoids.

**Additional file 2: Fig. S1.** Mouse MSC-CM activates the CFTR channel of intestinal epithelial cells. **Fig. S2.** PGE2 secreted by MSCs activates the CFTR channel of intestinal epithelial cells. **Fig. S3.** The PGE2 secretion of MSCs could be suppressed by COX inhibitors. **Fig. S4.** The expression of *Ptgs1* and *Ptgs2* in mouse colon. **Fig. S5.** COX deletion has minimal effects on the stool water content of mice. **Fig. S6.** *Muc2* expression is decreased in small intestinal crypts of *Gli1-Ptgs*^*cKO*^ mouse. **Fig. S7.** Deletion of COX1 and COX2 in mouse MSCs impairs cell proliferation. **Fig. S8.** *Muc2* expression is decreased in small intestinal of *Gli1-Ptgs*^*cKO*^ mouse after DSS treatment.

## Data Availability

The RNA-seq data have been made available at the Gene Expression Omnibus (GEO) repository under the accession number GSE243704: https://www.ncbi.nlm.nih.gov/geo/query/acc.cgi?acc=GSE243704. All the other data generated or analyzed during this study are included in this published article and its supplementary information files. Requests for materials should be addressed to the corresponding author.

## Abbreviations

MSCs: Mesenchymal stromal cells
PGE2: Prostaglandin E2
COX: Cyclooxygenase
cAMP: Cyclic adenosine monophosphate
CFTR: Cystic fibrosis transmembrane conductance regulator
IBD: Inflammation bowl disease
NSAIDs: Nonsteroidal anti- inflammatory drugs
CM: Conditional medium
PKA: Protein kinase A
scRNA-seq: Single-cell RNA sequencing
FACS: Fluorescence-activated cell sorting
DSS: Dextran sulphate sodium
DAI: Disease activity index
