# Correction: The cyclooxygenase-expressing mesenchyme resists intestinal epithelial injury by paracrine signaling

**DOI:** 10.1186/s13619-023-00178-3

**Published:** 2023-10-20

**Authors:** Siting Wei, Meng Li, Wanlu Song, Jiaye Liu, Shicheng Yu, Yalong Wang, Mengxian Zhang, Huijun Du, Yuan Liu, Huidong Liu, Wei Fu, Baojie Li, Ye-Guang Chen

**Affiliations:** 1 The State Key Laboratory of Membrane Biology, Tsinghua-Peking Center for Life Sciences, School of Life Sciences, Tsinghua University, Beijing, 100084 China; 2 Guangzhou National Laboratory, Guangzhou, 510005 China; 3 Department of Thyroid and Parathyroid Surgery, West China Hospital, Sichuan University, Chengdu, Sichuan China; 4 Department of General Surgery, Peking University Third Hospital, Beijing, China; 5 Bio-X Institutes, Key Laboratory for the Genetics of Developmental and Neuro-Psychiatric Disorders, Ministry of Education, Shanghai Jiao Tong University, Shanghai, 200240 China; 6 School of Basic Medicine, Jiangxi Medical College, Nanchang University, Nanchang, 330031 China

**Correction: Cell Regen 12, 30 (2023)**

**https://doi.org/10.1186/s13619-023-00174-7**

Following publication of the original article (Wei et al. 2023), it is reported that the “Availability of data and materials” section needs to be updated.

The original version was:

All data generated or analyzed during this study are included in this published article and its supplementary information files. Requests for materials should be addressed to the corresponding author.

The updated version is:

The RNA-seq data have been made available at the Gene Expression Omnibus (GEO) repository under the accession number GSE243704: https://www.ncbi.nlm.nih.gov/geo/query/acc.cgi?acc=GSE243704. All the other data generated or analyzed during this study are included in this published article and its supplementary information files. Requests for materials should be addressed to the corresponding author.

The original article (Wei et al. 2023) has been corrected.
