# Supplementary material for: The cyclooxygenase-expressing mesenchyme resists intestinal epithelial injury by paracrine signaling
Source: Cell Regen. 2023 Aug 14;12:30. doi: 10.1186/s13619-023-00174-7 (PMC10423710; doi:10.1186/s13619-023-00174-7)
Supplement: Supplementary file 2 — Additional file 2: Fig. S1. Mouse MSC-CM activates the CFTR channel of intestinal epithelial cells. Fig. S2. PGE2 secreted by MSCs activates the CFTR channel of intestinal epithelial cells. Fig. S3. The PGE2 secretion of MSCs could be suppressed by COX inhibitors. Fig. S4. The expression of Ptgs1 and Ptgs2 in mouse colon. Fig. S5. COX deletion has minimal effects on the stool water content of mice. Fig. S6. Muc2 expression is decreased in small intestinal crypts of Gli1-PtgscKO mouse. Fig. S7. Deletion of COX1 and COX2 in mouse MSCs impairs cell proliferation. Fig. S8. Muc2 expression is decreased in small intestinal of Gli1-PtgscKO mouse after DSS treatment. [file 13619_2023_174_MOESM2_ESM.docx]

**Supplementary information**

**The cyclooxygenase-expressing mesenchyme resists intestinal epithelial injury by paracrine signaling**

Siting Wei^1^†, Meng Li^2^†, Wanlu Song^1^, Jiaye Liu^3^, Shicheng Yu^2^, Yalong Wang^2^, Mengxian Zhang^1^, Huijun Du^1^, Yuan Liu^1^, Huidong Liu^1^, Wei Fu^4^, Baojie Li^5^ and Ye-Guang Chen^1, 2, 6^^*^

**Supplementary Figures 1-8**

**Supplemental Movie 1**


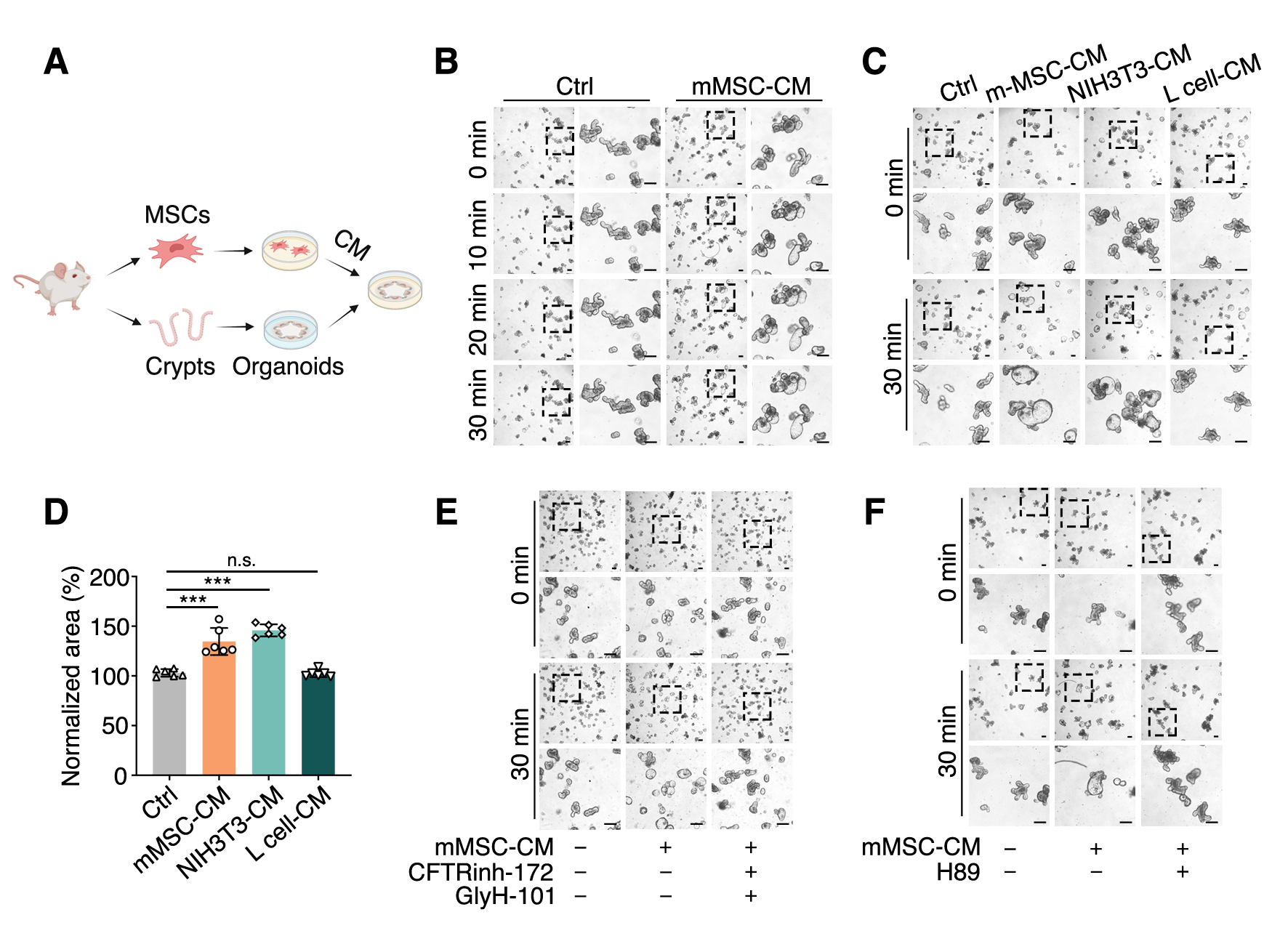


**Fig. S1 Mouse MSC-CM activates the CFTR channel of intestinal epithelial cells. (A)** Schematic diagram of co-culture of mouse small intestinal organoids and mouse MSC-CM. **(B)** Time-lapse imaging of mouse small intestinal organoids with mMSC-CM stimulation. Scale bar, 100 μm. **(C)** Time-lapse imaging of mouse small intestinal organoids with mMSC-CM, NIH3T3-CM or L cell-CM stimulation. Scale bar, 100 μm. **(D)** Surface area quantification of mouse small intestinal organoids with mMSC-CM, NIH3T3-CM or L cell-CM stimulation. **(E)** Time-lapse imaging of CFTRinh-172 and GlyH-101-treated mouse small intestinal organoids with mMSC-CM stimulation. The mouse small intestinal organoids were preincubated with 50 μM CFTRinh-172 and 50 μM GlyH-101 (CFTR inhibitors) for 3 h before stimulation. Scale bar, 100 μm. **(F)** Time-lapse imaging of H89-treated mouse small intestinal organoids with mMSC-CM stimulation. The mouse small intestinal organoids were preincubated with 10 μM H89 (PKA inhibitor) for 6 h before stimulation. Scale bar, 100 μm. Data represent mean ± SD of three independent experiments. ****P* < 0.001, n.s. not significant, one-way ANOVA (D).


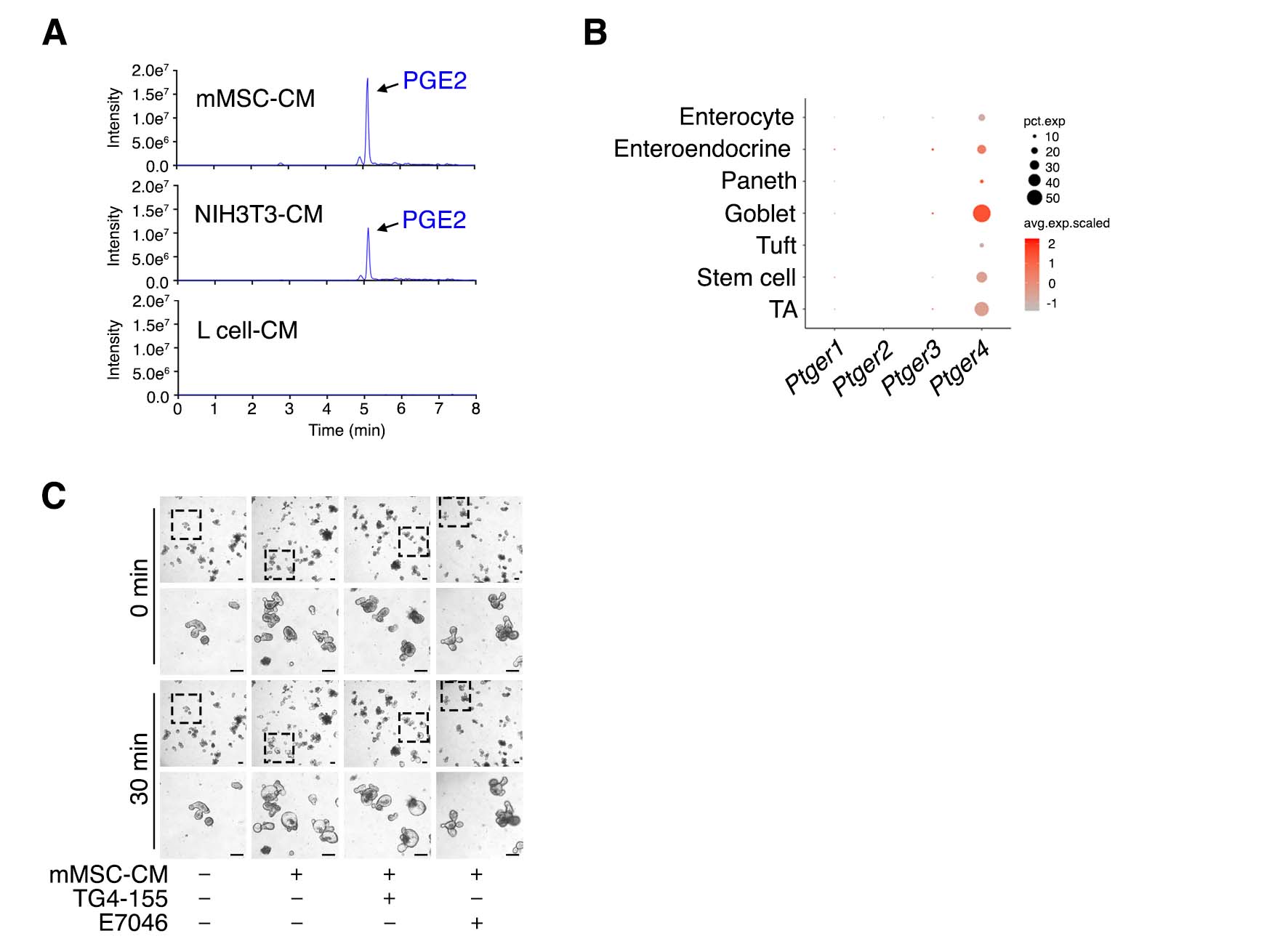


**Fig. S2 PGE2 secreted by MSCs activates the CFTR channel of intestinal epithelial cells. (A)** PGE2 intensity of mMSC-CM, NIH3T3-CM and L cell-CM, as revealed by LC-MS. **(B)** Dot plot showing the expression pattern of PGE2 receptors (*Ptger1, Ptger 2, Ptger 3, Ptger 4* encoding EP1, EP2, EP3, EP4 respectively) in subtypes of mouse small intestinal epithelial cells, as indicated by scRNA-seq. **(C)** Time-lapse imaging of E7046 or TG4-155-treated mouse small intestinal organoids with mMSC-CM stimulation. The mouse small intestinal organoids were preincubated with 10 μM E7046 (EP4 inhibitor) or 10 μM TG4-155 (EP2 inhibitor) for 72 h before stimulation. Scale bar, 100 μm.


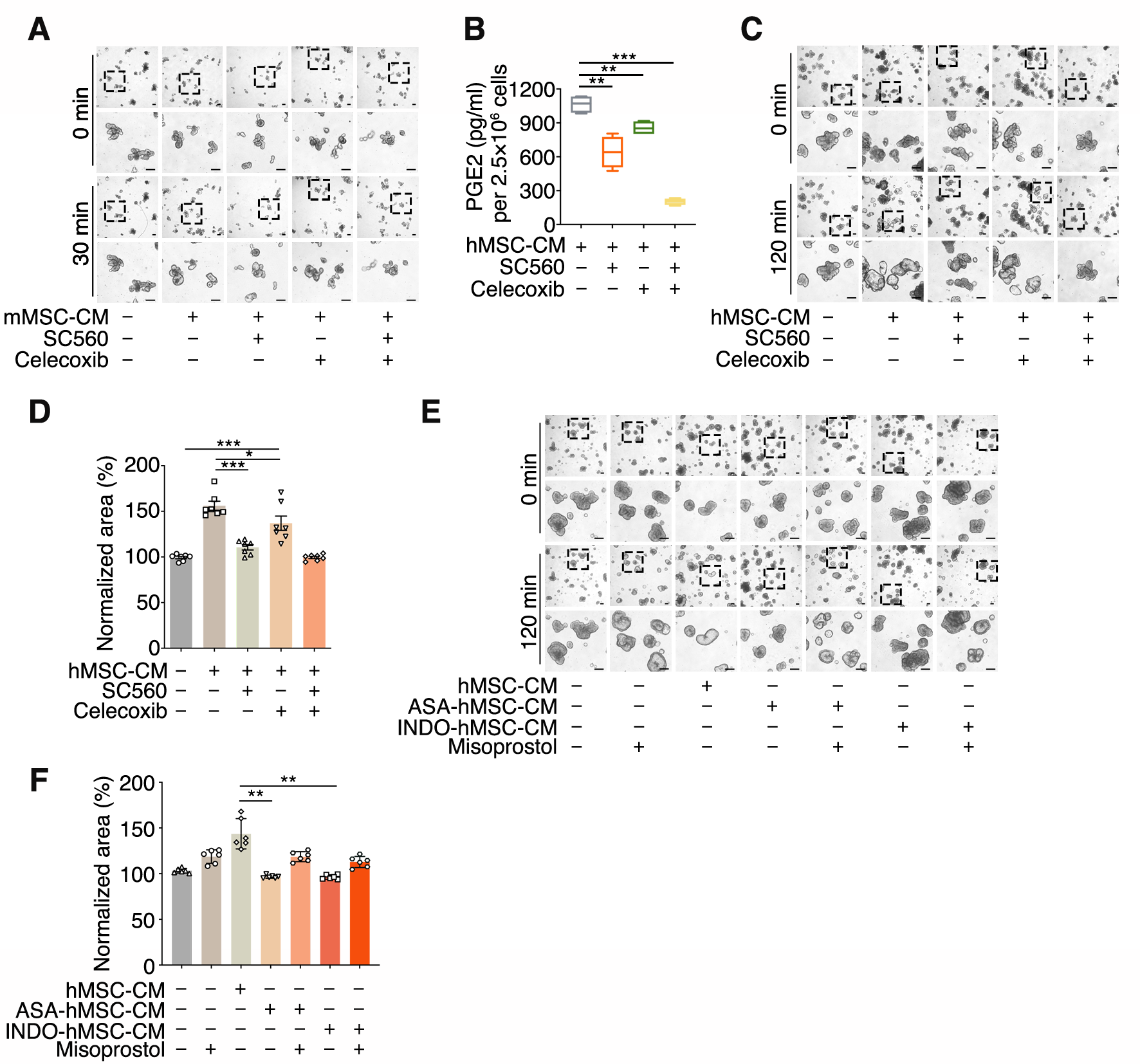


**Fig. S3 The PGE2 secretion of MSCs could be suppressed by COX inhibitors. (A)** Time-lapse imaging of mouse small intestinal organoids after stimulation using SC560 or Celecoxib-treated mMSC-CM. The mMSCs were preincubated with 20 nM SC560 (COX1 inhibitor), 150 nM Celecoxib (COX2 inhibitor), or both for 48 h, then the CM was collected for stimulation. Scale bar, 100 μm. **(B)** The PGE2 concentration of SC560 or Celecoxib-treated hMSC-CM, was quantified by ELISA assay. **(C, D)** Time-lapse imaging **(C)** and surface area quantification **(D)** of human ileum organoids after stimulation using SC560 or Celecoxib-treated hMSC-CM. The hMSCs were preincubated with 20 nM SC560, 150 nM Celecoxib, or both for 48 h, then the CM was collected for stimulation. Scale bar, 100 μm. **(E, F)** Time-lapse imaging **(E)** and surface area quantification **(F)** of human ileum organoids with hMSC-CM, ASA-hMSC-CM, INDO-hMSC-CM or misoprostol stimulation. ASA-hMSC-CM was collected from the hMSCs preincubated with aspirin (500 μM) for 48 h. INDO-hMSC-CM was collected from the hMSCs preincubated with indomethacin (50 μM). Misoprostol (20 μM) was used to induce the organoids swelling (t = 120 min). Scale bar, 100 μm. Data represent mean ± SD of three independent experiments. **P* < 0.05, ***P* < 0.01, ****P* < 0.001, one-way ANOVA (B, D, F).


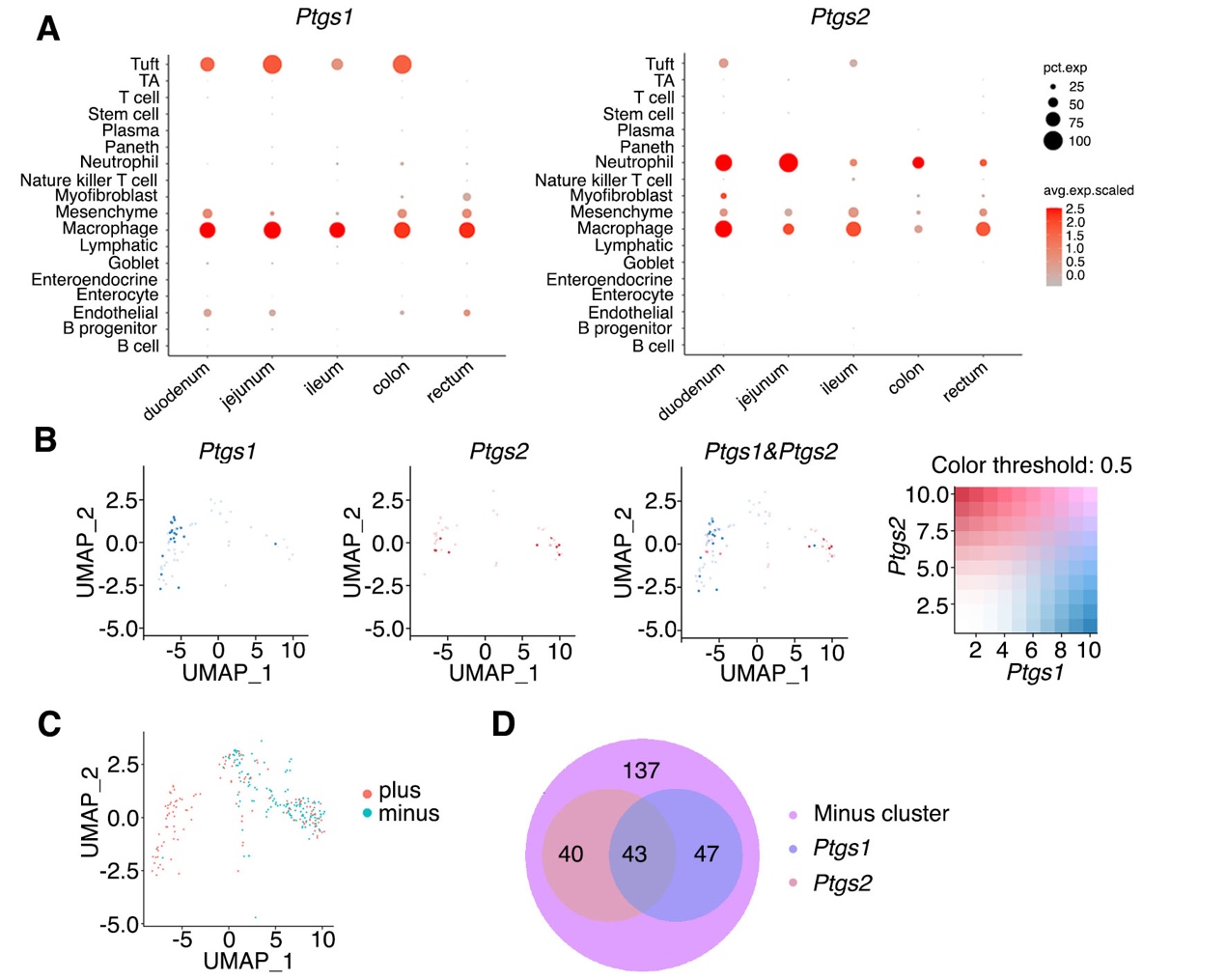


**Fig. S4 The expression of *Ptgs1* and *Ptgs2* in mouse colon. (A)** Dot plot showing the expression of *Ptgs1* (encoding COX1) and *Ptgs2* (encoding COX2) in subtypes of mouse colon, as revealed by scRNA-seq. **(B)** UMAP visualization of MSCs expressing *Ptgs1* or *Ptgs2* in mouse colon, as indicated by scRNA-seq. **(C)** UMAP visualization of plus and minus MSCs in mouse colon, as revealed by scRNA-seq. Plus, MSCs expressing either COX1 or COX2; Minus, MSCs expressing neither COX1 nor COX2. **(D)** Proportions of COX-expressing MSCs in total intestinal MSCs.


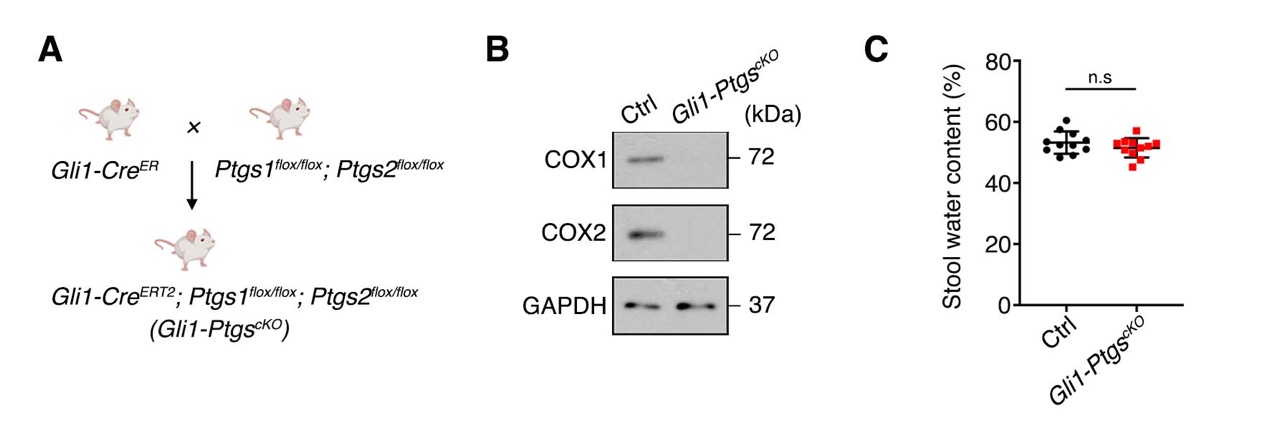


**Fig. S5 COX deletion has minimal effects on the stool water content of mice. (A)** Generation of *Gli1-Ptgs^cKO^* (*Gli1-Cre^ERT2^; Ptgs1^flox/flox^; Ptgs2^flox/flox^*) mouse strain. **(B)** Immunoblotting verifying the knockout efficiency of COX1 and COX2 in mMSCs. GAPDH served as a loading control. **(C)** The stool water content of *Gli1-Ptgs^cKO^* and Ctrl mice (*n* = 11 per genotype). Data represent mean ± SD of three independent experiments. ****P* < 0.001, unpaired two-tailed *t*-test (C).


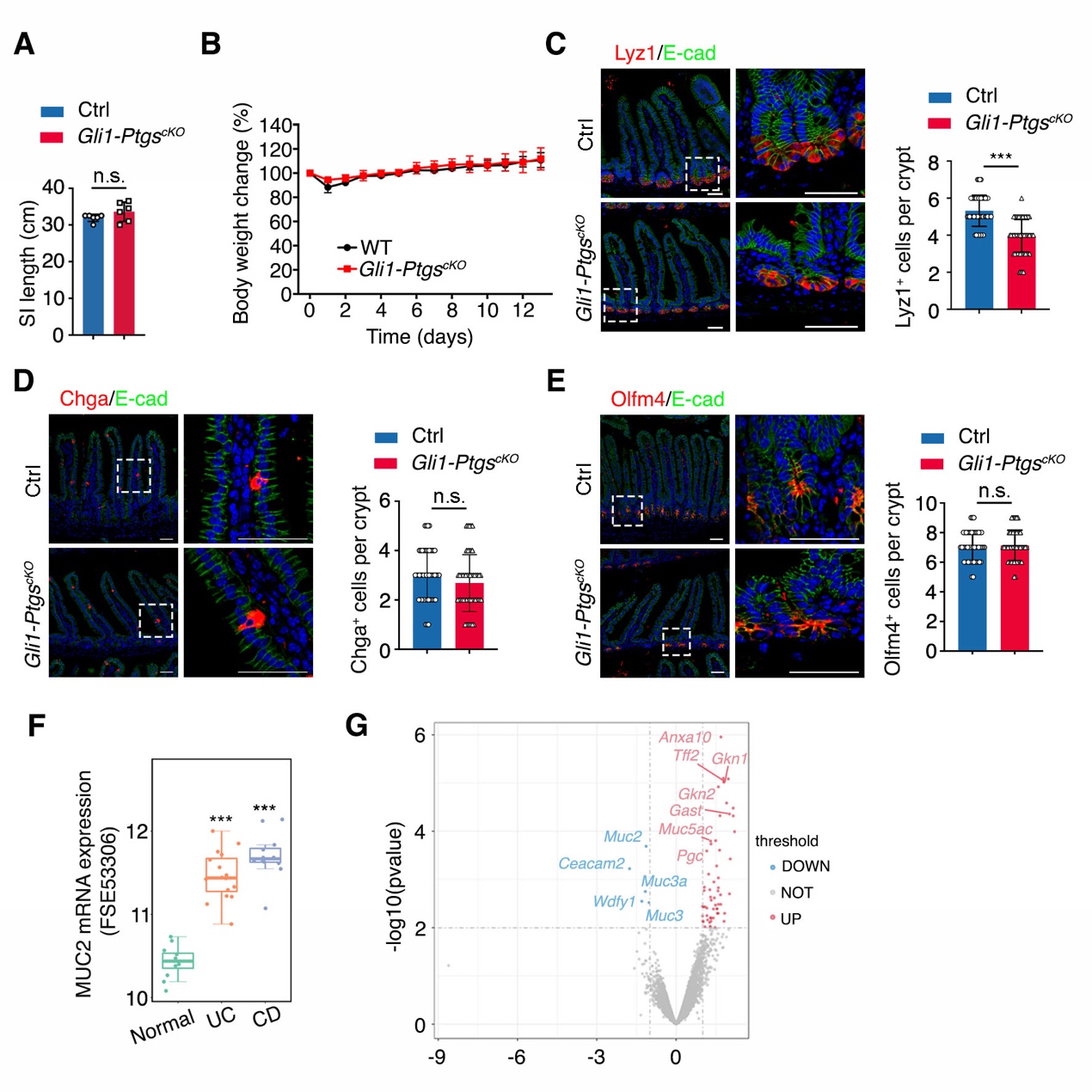


**Fig. S6 *Muc2* expression is decreased in small intestinal crypts of *Gli1-Ptgs^cKO^* mouse. (A)** The length analysis of small intestines (SI) from *Gli1-Ptgs^cKO^* and Ctrl mice. **(B)** Body weight changes of *Gli1-Ptgs^cKO^* and Ctrl mice after tamoxifen treatment. **(C)** Immunofluorescent staining of *Lyz1* and quantification of Lyz1^+^ cell number of *Gli1-Ptgs^cKO^* and Ctrl mouse small intestine. Scale bar, 100 μm. **(D)** Immunofluorescent staining of *Chga* and quantification of Chga^+^ cell number of *Gli1-Ptgs^cKO^* and Ctrl mouse small intestine. Scale bar, 100 μm. **(E)** Immunofluorescent staining of *Olfm4* and quantification of Olfm4^+^ cell number of *Gli1-Ptgs^cKO^* and Ctrl mouse small intestine. Scale bar, 100 μm. **(F)** Box plots of mRNA level of Muc2 in healthy controls and IBD specimens (using dataset GSE53306). The middle line depicts the median and the whisker depicts the min-to-max range. **(G)** Volcano map showing differential expression genes of small intestinal crypts between *Gli1-Ptgs^cKO^* and Ctrl mice, analyzed from bulk RNA-seq. Data represent mean ± SD of three independent experiments. ****P* < 0.001, unpaired two-tailed *t*-test (A, C, D, E), one-way ANOVA (F).
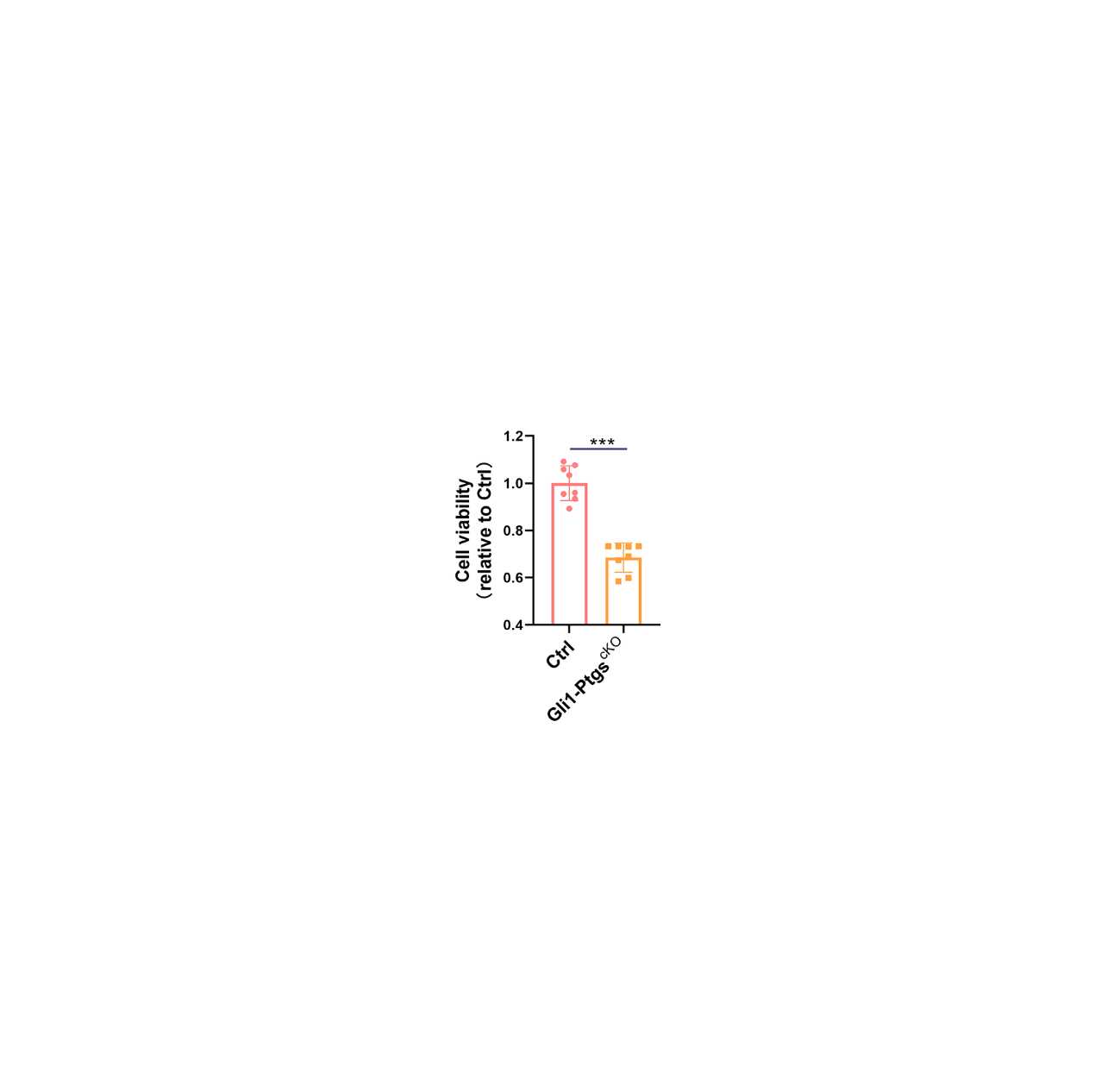


**Fig. S7 Deletion of COX1 and COX2 in mouse MSCs impairs cell proliferation. (A)** Cell viability of MSCs derived from *Gli1-Ptgs^cKO^* and control mice was measured by CCK8 kit. Data represent mean ± SD of three independent experiments. ****P* < 0.001, unpaired two-tailed *t*-test.


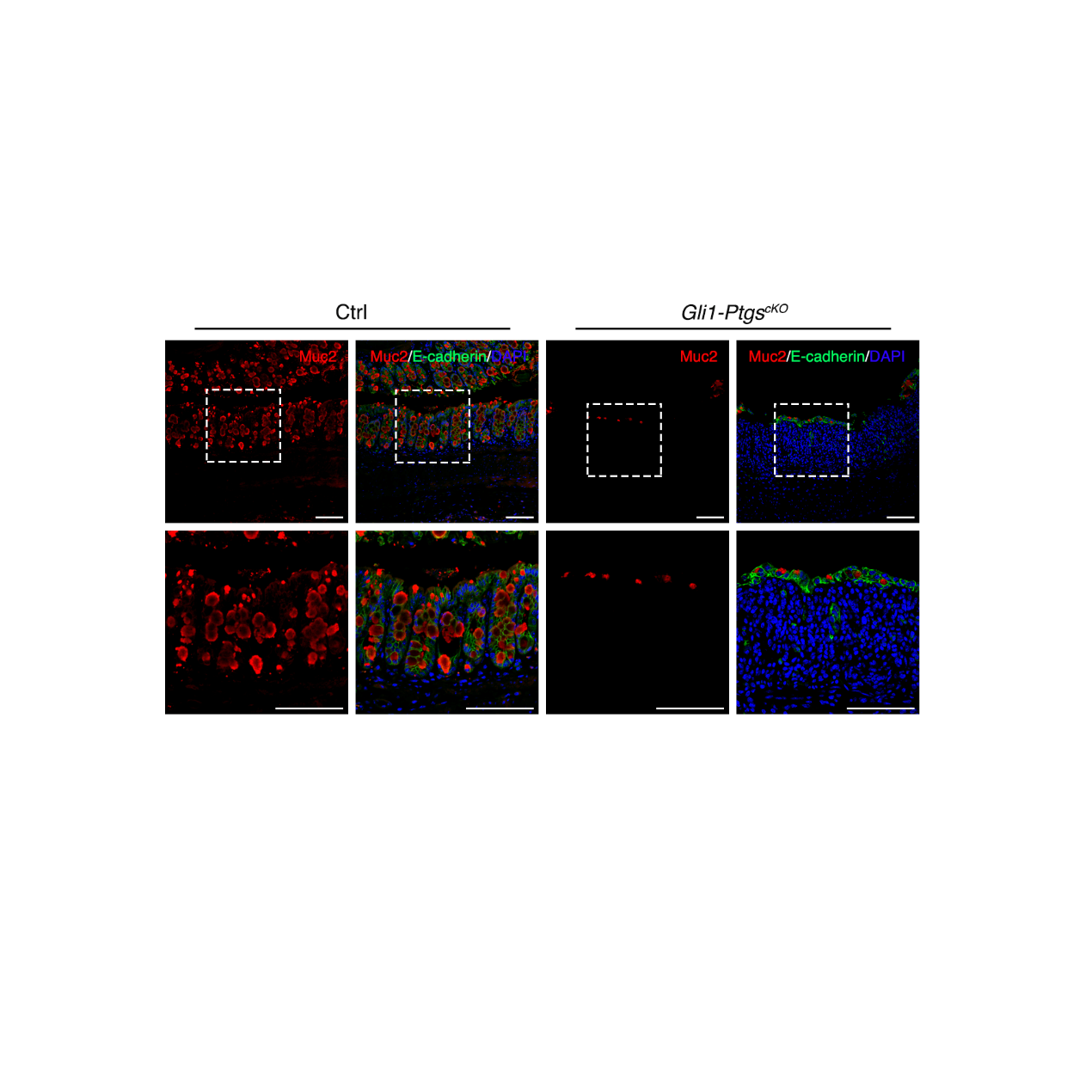


**Fig. S8 *Muc2* expression is decreased in small intestinal of *Gli1-Ptgs^cKO^* mouse after DSS treatment.** Immunofluorescent staining of *Muc2* and *E-cadherin* in *Gli1-Ptgs^cKO^* and cintrol mouse small intestine after 3% DSS treatment for 5 days. Scale bar, 100 μm.

**Supplemental Movie 1: Video shows mMSC-CM-induced swelling of mouse small intestinal organoids.** The organoids derived from *Apoa1-mCherry; Lgr5-EGFP* mice were coculture with mMSC-CM. Video shows the morphological change of *Apoa1-mCherry; Lgr5-EGFP* organoids over time from 0 min to 40 min.
